# Radiosensitivity in Non-Small-Cell Lung Cancer by MMP10 through the DNA Damage Repair Pathway

**DOI:** 10.1155/2023/5636852

**Published:** 2023-03-03

**Authors:** Yawei Bi, Kun Cao, Yuan Wang, Wei Yang, Na Ma, Xiao Lei, Yuanyuan Chen

**Affiliations:** ^1^Department of Gastroenterology and Hepatology, The First Medical Center of PLA General Hospital, Beijing 100859, China; ^2^Department of Radiation Medicine, Faculty of Naval Medicine, Naval Military Medical University, Shanghai 200433, China; ^3^Department of Radiation Oncology, Chinese PLA General Hospital, Beijing 100859, China

## Abstract

NSCLC (non-small-cell lung cancer) is an aggressive form of lung cancer and accompanies high morbidity and mortality. This study investigated the function and associated mechanism of MMP10 during radiotherapy of NSCLC. MMP10 expression in patients and their overall survival rate were assessed through GEPIA. Protein expression was tested by western blotting. Radioresistance was detected *in vitro* by apoptosis and clonogenic assay. The extent of DNA damage and repair was revealed by the comet test and *γ*H2AX foci test. High MMP10 levels in specimens of lung adenocarcinoma were related to poor patient outcomes. Clonogenic and apoptosis assays revealed that MMP10 knockdown in A549 cells initiated radiosensitization. Furthermore, MMP10 siRNA increased damage to the DNA in NSCLC cells, while MMP10 was observed to participate in DNA damage repair post-ionizing radiation. Thus, after irradiation, MMP10 plays an essential role in NSCLC through the repair pathway of DNA damage; regulating MMP10 for NSCLC radiosensitivity might have potential treatment implications in radiotherapy of NSCLC.

## 1. Introduction

NSCLC (non-small-cell lung cancer) is an aggressive lung cancer type that accompanies increased death rates and morbidity, including squamous cell carcinoma, large cell carcinoma, and adenocarcinoma [1]. The three methods commonly used for lung cancer treatment involve radiation, surgery, and chemotherapy [2]. However, over recent years, the development of “precision radiotherapy” is defined as stereotactic body radiation therapy, has indicated its precise, low, and noninvasive side effects, which furnishes more possibilities of treatment for lung cancer by radiotherapy [3]. While several methods for comprehensive radiotherapy-based treatment are involved in NSCLC, it tolerates ionizing radiation with progressive radiotherapy, indicating that most such patients had essentially serious effects [4–6].

MMP10 (matrix metalloproteinase-10) is an essential member of the MMP (matrix metalloproteinase) family [7]. It is a mesenchymal lysing enzyme that can break down the core collagen IV, V, IX, X-proteins, fibronectin, laminin, elastin, gelatin, and proteoglycan [8]. Because MMP10 has roles in several pathological and physiological processes, it is essential for tissue damage repair, embryonic development, and other processes [9, 10]. MMPs function in extracellular matrix (ECM) degradation and breakdown of the basement membrane tissues to facilitate tumor invasion, growth, and metastasis; besides, mediation of the ECM basement membrane is a significant stage for the transfer of tumors [11, 12]. While several studies on MMP-2 and MMP-9 have been reported, there are only a few reports on MMP10 and tumor associations [13, 14]. Recently, MMP10 was shown to play a significant role in pro-MMPs activation [15]; it is expressed at high levels in epithelial tumors like bladder transitional cell cancer, gastric cancer, esophageal cancer, NSCLC, and skin cancer [16–19]. These findings indicate a close relationship between MMP10 and the development and occurrence of tumors.

In this research, we examined the function of MMP10 in NSCLC and observed that MMP10 conferred resistance to radiotherapy in NSCLC via the repair pathway for the damaged DNA. The regulatory function of MMP10 on NSCLC radiosensitivity might have therapeutic possibilities in the radiotherapy of NSCLC.

## 2. Materials and Methods

### 2.1. Public Bioinformatics Analysis

Differentially genes were obtained using the limma R package from TCGA-LUAD, TCGA-LUSC, and normal lung tissues (GTEx). Here, MMP10 (NM_002425.3) expression between tumorous tissues of LUAD, LUSC, and normal surrounding tissues was analyzed using the “Expression DIY” module of GEPIA [20]. Survival analysis was performed according to the MMP10 expression status and Kaplan–Meier curves were plotted; comparison of MMP10 mutations was done according to the survival status (LIVING/DECEASED) in 514 TCGA-LUAD patients using cBioportal (https://cbioportal.org). The Spearman method was used for the expression correlation between DDR-related genes and MMP10.

### 2.2. Cell Culture and Treatment

A549, the human LUAD (lung adenocarcinoma) cell line, was procured from ATCC (USA). They were cultured in DMEM containing fetal bovine serum (10%) at 37°C in an incubator with a CO_2_ (5%) chamber with appropriate humidity. A549 cells were radiated at dose of 8 Gy (clonogenic assay with 0 Gy, 2 Gy, 4 Gy, and 8 Gy). For apoptosis assay, the cells were detected by flow cytometry 24 h after radiation. Cellular state and density were observed during culture and fluid was changed on alternate days.

### 2.3. Irradiation

For cell radiation treatment, we used ^60^Co *γ*-rays (Radiation Center, Faculty of Naval Medicine of the Second Military Medical University, Shanghai, China). A specific dose was given to the cells at a rate of 1 Gy/min. All irradiations were performed at room temperature.

### 2.4. siRNA and Cellular Transfections

MMP10 siRNA was obtained from Thermo Fisher (Cat.^#^AM16708). MMP10 siRNA was transfected along with lipofectamine 3,000 from Invitrogen as per the provided instructions. Cells transfected with the empty vector were used as negative control (NC), along with untransformed cells (parental). A549 cells were cultured for at least 24 h after transfection and then exposed to radiation. Cells that were successfully transfected were used for assays at specified time points.

### 2.5. Clonogenic Assay

A549 cell survival was examined by clonogenic assay. The cells were trypsinized, counted, and seeded in 60-mm culture dishes in two sets of three for each dose of radiation; the number of cells seeded was according to the dose of radiation (0 Gy-200 cells, 2 Gy-400 cells, 4 Gy-800 cells, and 8 Gy-1600 cells), followed by irradiation with 0, 2, 4, and 8 Gy after 24 h. After ten days, cells were fixed using paraformaldehyde and methylene blue (1%) stain. Thirty minutes later, dishes were washed using phosphate-buffered saline (PBS) and dried naturally. Then, the clone formation was counted.

### 2.6. Apoptosis Assay

To stain the irradiated A549 cells, Annexin V-fluorescein isothiocyanate (AV) and propidium iodide (PI) in the kit for apoptosis detection from Invitrogen (California, USA) were utilized. The cells were plated in six-well plates at a density of 10^5^ cells per well and allowed to attach for 24 h. 24 h after 8 Gy radiation, the cells were harvested by trypsin digestion, washed with precooled phosphate-buffered saline (PBS) twice, and resuspended. Then, the cells were stained with AV and PI at room temperature for 15 min in a dark room. Flow cytometry (Beckman CytoFLEX) was conducted for analyses as per the instructions of the manufacturer.

### 2.7. Neutral Comet Assay

The extent of damage to DNA of A549 cells was examined by the neutral comet assay using a kit from Trevigen Inc. (Gaithersburg, MD) that was used at 4 h and 8 h post-irradiation as per the protocol provided by the manufacturer. First, slides were immersed in a 1% NMA and dry thoroughly. Next, the single cell suspension prepared (2 × 10^4^ cells/ml) was immersed in LMA under a 40°C water bath. Third, cell suspension was mixed and rapidly pipetted onto the surface of the precoated slide. The slides were then incubated at 4°C for 25 min at 25 V in TBE. Then, the gel was stained with PI (10 *μ*g/ml) for 20 min and then rinsed gently with ddH2O. Finally, all slices were examined by an Olympus BX60 fluorescence microscope. Total 100 images in each slide were analyzed using CASP 1.2.3b2 software (CASPlab, Poland).

### 2.8. Western Blotting

Post-irradiation, at 0 h, 0.5 h, and 8 h, preparation of total cell lysates was performed using the ProtectJETTM Mammalian Cell Lysis Reagent from Fermentas (Lithuania) as per the protocol provided by the manufacturer. The membranes with the transferred protein were incubated with gentle agitation with following specific primary antibodies (1 : 1,000) at 4°C overnight: p-ATM (1 : 1000), p-DNA-PKcs (1 : 1000), Rad51 (1 : 1000), MMP10 (1 : 1000), and actin (1 : 1000) (all primary antibodies were from Abcam, USA). The secondary antibody (1 : 5000) were also from Abcam. Electrochemiluminescence (Santa Cruz Biotechnology Inc) was used to detect all the membranes.

### 2.9. Immunofluorescence Staining

For this, *γ*H2AX foci, a marker for DNA double-strand breaks, was detected via immunofluorescence assay. Post-2 Gy radiation, transfection of A549 cells with siRNA against MMP10 was conducted. At specified times, cells were fixed with chilled methanol/acetone (1 : 1); then, BSA (3%; in PBS) was used for blocking at room temperature for 60 min. Then, cells allowed to bind to a *γ*H2AX primary antibody (1 : 300; Abcam, US) were reacted with the secondary antibody (1 : 1000). Then, confocal and conventional microscopy was used to monitor immunofluorescence; each group recorded the number of *γ*H2AX foci in 30 cells and took the average.

### 2.10. Statistical Analysis

Data were acquired after conducting a minimum of three experiments conducted independently and were presented as the mean ± standard deviation. The statistical significance limit was considered to be *P* <  0.05.

Per treatment, for all the experimental groups, mean and standard error (SEM) was calculated. For all pairwise comparison procedures, Student's *t*-test was used, including calculating *P* values.

## 3. Results

### 3.1. High Levels of MMP10 in Lung Adenocarcinoma Correlates with Poor Patient Outcomes

**Figure 1:**
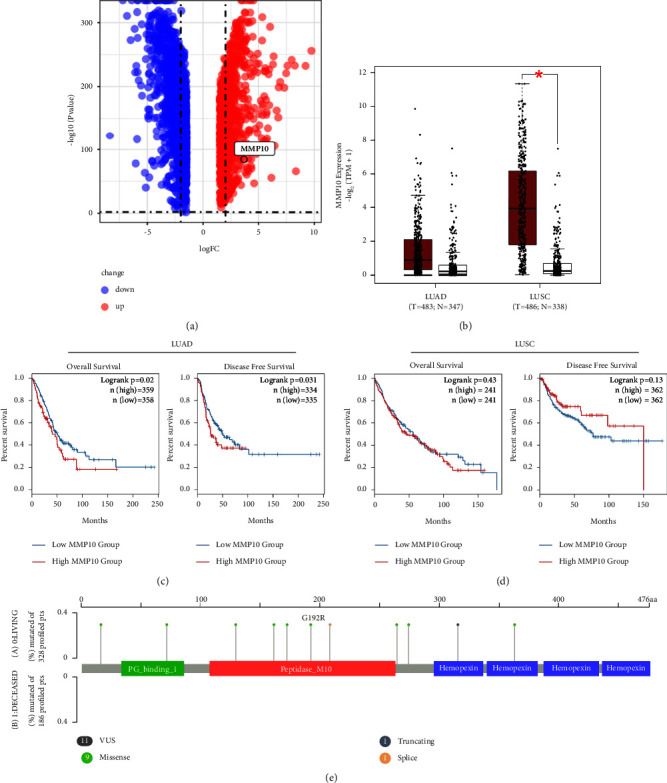
High expression of MMP10 in specimens of LUAD is related to poor patient outcomes. (a, b) MMP10 expression in NSCLC as per TCGA and GTEx sample types. (c, d) The impact of MMP10 level on the survival of LUAD/LUSC patients (overall survival and disease free survival). (e) Analysis of clinical data in TCGA-LUAD combined with MMP10 gene mutation data. (LUAD, lung adenocarcinoma; LUSC, lung squamous cell carcinoma).

We analysed the expression of MMP10 in both LUAD and LUSC specimens by R-language according to TCGA and GTEx databases. The expression of MMP10 in the two types of NSCLC was higher than that in normal lung tissue, but only the expression of MMP10 in LUSC was statistically significant (Figure 1(a)). The MMP10 level in 486 primary LUAD specimens (Figure 1(b)) was remarkably higher than 338 healthy tissues. We then explored the relationship between MMP10 levels and LUAD/LUSC patient lifespan through R-language. Overall survival and disease-free survival were significantly lower for patients with MMP10^high^ LUAD (Figures 1(c) and 1(d)) relative to those with MMP10^low^ tumors (*P* < 0.05). Then, we analyzed the clinical data combined with MMP10 gene mutation data in TCGA-LUAD via cBioPortal (https://cbioportal.org) online tools. The results show that a total of 11 mutation sites (including 9 Missense, 1 Truncating, and 1 Splice) were found between 0 and 476 amino acids of MMP10 and 9 mutations in the domain. These mutations were all concentrated in the previous LIVING group, and none of the 186 cases in the DECEASED group had mutations, which illuminated the prognosis of LUAD patients with MMP10 mutation that shows better survival level (Figure 1(e)).

### 3.2. Impact of MMP10 siRNA on A549 Cell Survival and Apoptosis Post-Irradiation

**Figure 2:**
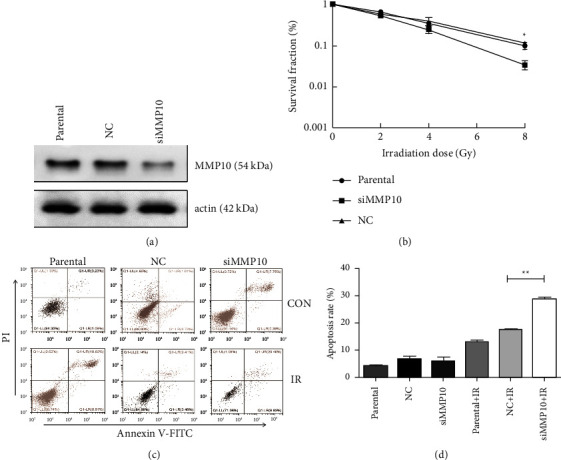
Impact of MMP10 siRNA on A549 cell survival and apoptosis post-IR. (a) Level of MMP10 expression in A549 cells was determined by western blot assay; for loading control, actin was used. (b) A549 and its MMP10 knockdown cell lines were examined for their ability to form colonies against IR. (c) Flow cytometry analysis of apoptosis in A549 and its MMP10 knockdown cell lines against IR. (d) A column chart presenting flow cytometric assessment of A549 cell line against IR in the presence/absence of MMP10 siRNA. (NC, negative control) ^*∗*^*P* < 0.05, ^*∗∗*^*P* < 0.01.

To reveal the effects of MMP10 in radio treatment, we first used MMP10 siRNA for inhibiting the expression of MMP10 in A549 cells (Figure 2(a)). Then, using these cellular models for clonogenic assay, MMP10 knockdown rendered these cells significantly sensitive to IR (Figure 2(b)). Furthermore, we explored the effect of siMMP10 on the apoptosis of A549 cell after irradiation using flow cytometry. As we could see in Figures 2(c) and 2(d), although there were little differences in early apoptotic rate (fourth quadrant) and late apoptotic rate (first quadrant) between group parental and group NC (negative control) due to little difference in detection time, the total number of apoptosis (first and fourth quadrant) detected in the siMMP10 group was significantly less than that in groups parental and NC, which meant MMP10 knockdown significantly promotes A549 cell apoptosis post-IR.

### 3.3. Increase in NSCLC Cell DNA Damage Due to MMP10 siRNA Post-IR

**Figure 3:**
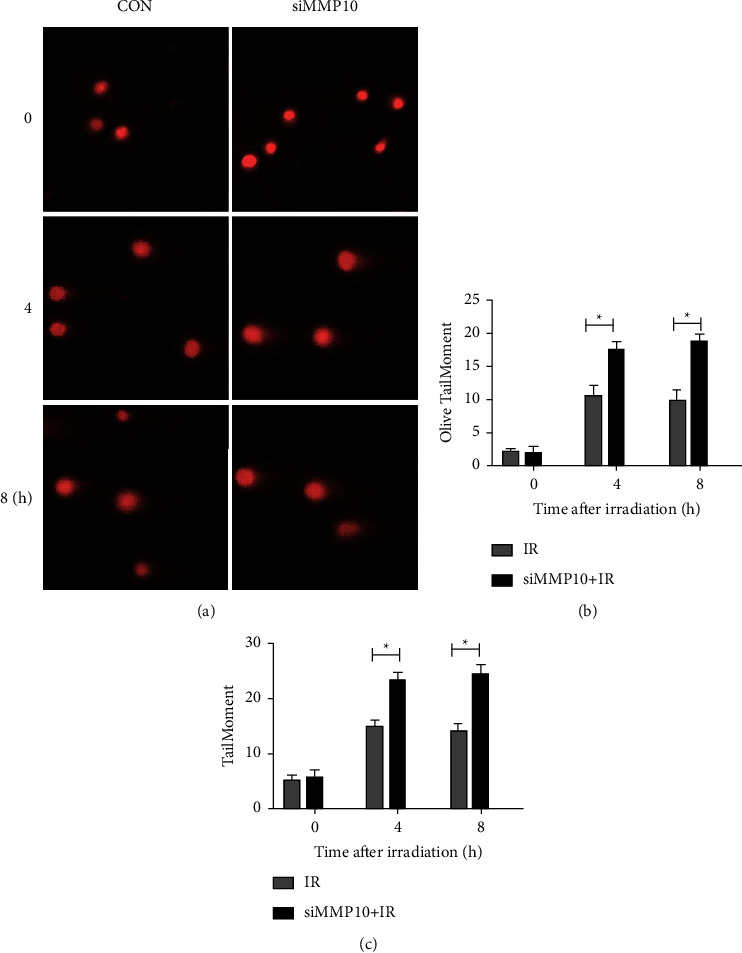
Increase in NSCLC cell DNA damage due to MMP10 siRNA treatment post-IR. (a) Representative images of comet assay revealing A549 cells tail movement after IR exposure (*n* = 3 experiments conducted independently). Quantification results are presented in (b) and (c) (data represent the mean ± SEM). ^*∗*^*P* < 0.05.

Then, comet assay was conducted to reveal the activity of MMP10 in NSCLC post-IR and examine the extent of DNA damage using MMP10 siRNA. Indeed, MMP10 expression knockdown enhanced the damage to DNA post-IR (Figures 3(a)–3(c)), suggesting a possible, important function of MMP10 in the repair pathway for DNA damage post-IR.

### 3.4. Involvement of MMP10 in the Pathway for DNA Damage Repair

**Figure 4:**
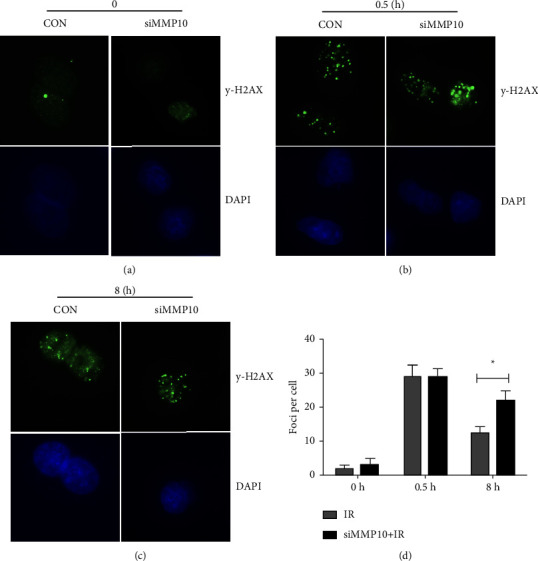
Impairment of the repair process of the damaged DNA to MMP10 knockdown. (a–c) Representative images showing foci of *γ*H2AX at specified times after 2 Gy radiation. (d) Column chart of the analysis of *γ*H2AX foci assay in A549 cell line against IR. ^*∗*^*P* < 0.05.

**Figure 5:**
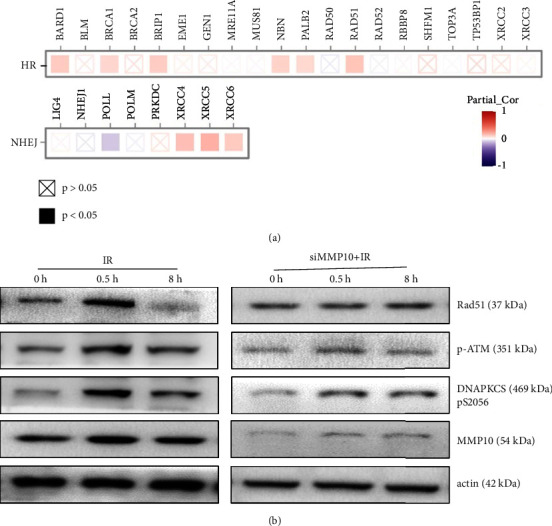
The involvement of MMP10 in the pathway for DNA damage repair. (a) Correlation between core genes of DDR pathway (HR and NHEJ) and expression of MMP10 in LUAD. (b) Western blot to examine proteins involved in DNA damage repair in A549 cells in different groups. (HR, homologous recombination; NHEJ, nonhomologous end-joining).

For confirming our inference, we examined the pathway for DNA damage repair by using western blot and immunofluorescence post-IR and treatment with MMP10 siRNA. The *γ*H2AX foci assay revealed a much higher foci number at 8 h after IR in the siMMP10 + IR group than in the IR group (Figures 4(a)–4(d)), suggesting significant impairment of DNA repair as a result of MMP10 knockdown in response to IR. DNA damage repair in body after radiation is mainly carried out through NHEJ (nonhomologous end-joining) and HR (homologous recombination) pathways. Among them, HR is a completely correct repair pathway, because it requires homologous sister chromatids as templates, so the repair process only occurs during the S and G2 phases of DNA replication. In contrast, the NHEJ pathway plays roles throughout the cell cycle because it can directly rejoin broken DNA without the need for homologous sequences, and thus is often the primary repair modality for DSBs [21]. Related studies have shown that the DNA damage repair mechanism in tumor cells is extremely active, and a series of DNA damage repair-related proteins (ATM, DNA-PKcs, and Rad51) are involved in the regulation of tumor radiation resistance [22]. In order to further explore the relationship between MMP10 and DNA damage repair pathway, we tested the correlation between core genes of DDR pathway (HR and NHEJ) and expression of MMP10 in LUAD, which we found partial core genes of the DDR pathway were positively correlated with MMP10 expression, especially the genes in the HR pathway (Figure 5(a)). Further examination showed the inhibition of phosphorylation of proteins involved in DNA damage repair post-MMP10 siRNA treatment (Figure 5(b)), indicating the involvement of MMP10 in the pathway for DNA damage repair post-IR.

## 4. Discussion

This study revealed the involvement of MMP10 in NSCLC radiosensitivity through the pathway for DNA damage repair. First, R-language was used to reveal a significantly high expression of MMP10 in LUAD samples as per TCGA and GTEx samples, relative to that in normal tissue. Next, analysis of the correlation between the expression of MMP10 with LUAD patient lifespan revealed significantly lower rates of overall survival for patients with MMP10^high^ LUAD than those having tumors with MMP10^low^ (*P* < 0.05). We also found that MMP10 gene mutation data in TCGA-LUAD showed a better survival level which meant MMP10 might play a role in promoting tumor progression indirectly. IR can induce double-stranded DNA breaks and the subsequent apoptosis of corresponding cells [23]. Based on the bioinformatics analysis results, next, we used siMMP10 on A549 cells which rendered radioresistance for a better cell survival rate and lower apoptosis rate compared with negative control. Besides, we found MMP10 was closely associated with DNA damage through neutral comet assay. This brought us great interest so that we did series of experiments to detect the relationship between MMP10 and DNA damage repair after IR. Then, we illuminated that the knockdown of MMP10 increased the damage to DNA post-IR through the inhibition of the pathway for DNA damage repair, which we deem the primary reason for resistance to radiotherapy in NSCLC.

Radio treatment is an important approach to the treatment of tumors in lung cancer, although the outcome is not so satisfactory [24]. Studies on radiosensitization involve the following aspects: tolerance of tumor cells to hypoxia, repair of damaged DNA, apoptosis, angiogenesis, and disorders of the cell cycle [25–27]. While research on radiosensitization has progressed, it is still in the preliminary stage. With the resistance to radiotherapy of lung cancer cells, several uncertainties still exist in the treatment [28, 29].

MMP10, as an essential component of the MMP family, is active in various pathological and physiological processes and is essential for the repair of the damaged tissue, development of the embryo, and other processes [30]. Recently, MMP10 was found to be essential for pro-MMP activation [31]; a high expression of MMP10 was observed in tumors epithelial cells, such as transitional cell cancer of the bladder, gastric cancer, skin cancer esophageal cancer, and NSCLC [17, 32–34]. In this study, we observed that MMP10 knockdown significantly inhibited A549 cell survival and facilitated apoptosis post-IR. Furthermore, MMP10 knockdown could enhance the extent of DNA damage post-IR. Studies have shown that abnormally active DNA damage repair ability is the core mechanism of tumor cell to resist IR, which is also the main reason why tumor cells have a better survival rate and lower apoptosis rate [35]. DNA strand breaks are severe damages caused by IR, which can be divided into DNA single-strand breaks, DNA double-strand breaks, DNA base damage, and DNA crosslinks. Among them, DNA double-strand breaks (DSBs) are the most important form of damage caused by IR, and it is also recognized as the most serious form of damage [36]. In response to DSBs, cells establish complex signaling networks for the activation of DNA damage checkpoints. Once the cell detects damage, a host of DNA repair factors localize to the site of chromatin damage and initiate the DNA repair machinery by recruiting other repair proteins. In eukaryotic cells, DSBs are mainly repaired by the NHEJ and HR pathways [37]. Therefore, we made an assessment of the repair pathway for DNA damage by western blotting and immunofluorescence suggested a crucial function of MMP10 in NSCLC post-radiation through the pathway for DNA damage repair and the regulatory role of MMP10 on NSCLC radiosensitivity may confer therapeutic indications to radiotherapy. In addition, as both the NHEJ and HR pathways proteins (DNA-PKcs, ATM, and Rad51) phosphorylation were reduced in our result; MMP10 might affect the upstream proteins of the NHEJ and HR pathways, which meant that MMP10 might be involved in the core regulation of DNA damage repair; this is our next research direction.

Although radiotherapy is currently the mainstay of NSCLC treatment, tumor radioresistance has greatly limited the efficacy of radiotherapy. As DNA strand breaks are the main reason for cell death caused by IR, screening and discovering the key molecules involved in DNA radiation damage repair and elucidating their mechanism are the core basic issues in the field of radiotherapy. But so far, there are very few genes that could be clinically targeted for radiosensitization. Our research results suggest that MMP10 may play an important role in tumor radioresistance. In the next step, we will continue to study how MMP10 regulates DNA damage repair pathway and carry out clinical transformation.

To conclude, this is the first report to show that knockdown MMP10 can significantly radiosensitize NSCLC. We also find that MMP10 regulates tumor radiosensitivity through the DNA damage repair pathway. These novel findings would possibly aid in discovering new mechanism to enhance radiosensitivity to NSCLC.

## Data Availability

The datasets are available under reasonable request.

## References

[1] Kunimasa K., Goto T. (2020). Immunosurveillance and immunoediting of lung cancer: current perspectives and challenges. *International Journal of Molecular Sciences*.

[2] Li X. Y., Lin J. Z., Yu S. H. (2020). Front-line therapy in advanced non-small cell lung cancer with sensitive epidermal growth factor receptor mutations: a network meta-analysis. *Clinical Therapeutics*.

[3] Tsao M. N. (2019). Stereotactic Body Radiation Therapy for Extracranial Oligometastatic Non-small-cell Lung Cancer: A Systematic Review. *Clinical lung cancer*.

[4] Garcia Molina E., Penas-Prado M. (2020). Neoplastic Meningitis in Solid Tumours: Updated Review of Diagnosis, Prognosis, Therapeutic Management, and Future Directions. *Neurologia*.

[5] Elmorabit B., Derhem N., Khouchani M. (2019). Plasmocytome solitaire pulmonaire traité par radiothérapie: à propos d’un cas et revue de la littérature. *The Pan African medical journal*.

[6] Liu L., Yang Y., Guo Q. (2020). Comparing hypofractionated to conventional fractionated radiotherapy in postmastectomy breast cancer: a meta-analysis and systematic review. *Radiation Oncology*.

[7] Solov’eva N. I. (1998). [Matrix metalloproteinases and their biological functions]. *Bioorganicheskaia Khimiia*.

[8] Razai A. S., Eckelman B. P., Salvesen G. S. (2020). Selective inhibition of matrix metalloproteinase 10 (MMP10) with a single-domain antibody. *Journal of Biological Chemistry*.

[9] Rohani M. G., Dimitrova E., Beppu A., Wang Y., Jefferies C. A., Parks W. C. (2018). Macrophage MMP10 regulates TLR7-mediated tolerance. *Frontiers in Immunology*.

[10] McMahan R. S., Birkland T. P., Smigiel K. S. (2016). Stromelysin-2 (MMP10) moderates inflammation by controlling macrophage activation. *The Journal of Immunology*.

[11] Faraj Shaglouf L. H., Ranjpour M., Wajid S., Jain S. K. (2020). Elevated expression of cellular SYNE1, MMP10, and GTPase1 and their regulatory role in hepatocellular carcinoma progression. *Protoplasma*.

[12] Upadhyay P., Gardi N., Desai S. (2017). Genomic characterization of tobacco/nut chewing HPV-negative early stage tongue tumors identify MMP10 asa candidate to predict metastases. *Oral Oncology*.

[13] Asghar M. Y., Kemppainen K., Lassila T., Tornquist K. (2018). Sphingosine 1-phosphate attenuates MMP2 and MMP9 in human anaplastic thyroid cancer C643 cells: importance of S1P2. *PLoS One*.

[14] Luo S., Huang G., Wang Z. (2015). Niflumic acid exhibits anti-tumor activity in nasopharyngeal carcinoma cells through affecting the expression of ERK1/2 and the activity of MMP2 and MMP9. *International Journal of Clinical and Experimental Pathology*.

[15] Rodriguez J. A., Sobrino T., Orbe J. (2013). proMetalloproteinase-10 is associated with brain damage and clinical outcome in acute ischemic stroke. *Journal of Thrombosis and Haemostasis*.

[16] Gobin E., Bagwell K., Wagner J. (2019). A pan-cancer perspective of matrix metalloproteases (MMP) gene expression profile and their diagnostic/prognostic potential. *BMC Cancer*.

[17] Xu J., E C., Yao Y., Ren S., Wang G., Jin H. (2016). Matrix metalloproteinase expression and molecular interaction network analysis in gastric cancer. *Oncology Letters*.

[18] Shi X., Chen Z., Hu X. (2016). AJUBA promotes the migration and invasion of esophageal squamous cell carcinoma cells through upregulation of MMP10 and MMP13 expression. *Oncotarget*.

[19] Justilien V., Regala R. P., Tseng I. C. (2012). Matrix metalloproteinase-10 is required for lung cancer stem cell maintenance, tumor initiation and metastatic potential. *PLoS One*.

[20] Tang Z., Kang B., Li C., Chen T., Zhang Z. (2019). GEPIA2: an enhanced web server for large-scale expression profiling and interactive analysis. *Nucleic Acids Research*.

[21] Vignard J., Mirey G., Salles B. (2013). Ionizing-radiation induced DNA double-strand breaks: a direct and indirect lighting up. *Radiotherapy & Oncology*.

[22] Huang R., Zhou P. K. (2021). DNA damage repair: historical perspectives, mechanistic pathways and clinical translation for targeted cancer therapy. *Signal Transduction and Targeted Therapy*.

[23] Chen Y., Zhao Y., Yang X. (2022). USP44 regulates irradiation-induced DNA double-strand break repair and suppresses tumorigenesis in nasopharyngeal carcinoma. *Nature Communications*.

[24] Chua K. L. M., Sin I., Fong K. W., Chua M. L. K., Onishi H. (2017). Stereotactic body radiotherapy for early stage lung cancer-historical developments and future strategies. *Chinese Clinical Oncology*.

[25] Howard D., Sebastian S., Le Q. V. C., Thierry B., Kempson I. (2020). Chemical mechanisms of nanoparticle radiosensitization and radioprotection: a review of structure-function relationships influencing reactive oxygen species. *International Journal of Molecular Sciences*.

[26] Kumar A., Becker D., Adhikary A., Sevilla M. D. (2019). Reaction of electrons with DNA: radiation damage to radiosensitization. *International Journal of Molecular Sciences*.

[27] Schulz A., Meyer F., Dubrovska A., Borgmann K. (2019). Cancer stem cells and radioresistance: DNA repair and beyond. *Cancers*.

[28] Lin J., Song T., Li C., Mao W. (2020). GSK-3beta in DNA repair, apoptosis, and resistance of chemotherapy, radiotherapy of cancer. Biochimica et biophysica acta. *Molecular cell research*.

[29] Farias V. d. A., Tovar I., del Moral R. (2019). Enhancing the bystander and abscopal effects to improve radiotherapy outcomes. *Frontiers in Oncology*.

[30] Mora-Gutierrez J. M., Rodriguez J. A., Fernandez-Seara M. A. (2020). MMP-10 is increased in early stage diabetic kidney Disease and can be reduced by renin-angiotensin system blockade. *Scientific Reports*.

[31] Vandivort T. C., Birkland T., Domiciano T., Mitra S., Kavanagh T., Parks W. (2017). Stromelysin-2 (MMP-10) facilitates clearance and moderates inflammation and cell death following lung exposure to long multiwalled carbon nanotubes. *International Journal of Nanomedicine*.

[32] Furuya H., Chan O. T., Hokutan K. (2019). Prognostic significance of lymphocyte infiltration and a stromal immunostaining of a bladder cancer associated diagnostic panel in urothelial carcinoma. *Diagnostics*.

[33] Schlage P., Kockmann T., Sabino F., Kizhakkedathu J. N., auf dem Keller U. (2015). Matrix metalloproteinase 10 degradomics in keratinocytes and epidermal tissue identifies bioactive substrates with pleiotropic functions∗. *Molecular & Cellular Proteomics*.

[34] Cooke M., Casado-Medrano V., Ann J. (2019). Differential regulation of gene expression in lung cancer cells by diacyglycerol-lactones and a phorbol ester via selective activation of protein kinase C isozymes. *Scientific Reports*.

[35] Goldstein M., Kastan M. B. (2015). The DNA damage response: implications for tumor responses to radiation and chemotherapy. *Annual Review of Medicine*.

[36] Carusillo A., Mussolino C. (2020). DNA damage: from threat to treatment. *Cells 9*.

[37] Groelly F. J., Fawkes M., Dagg R. A., Blackford A. N., Tarsounas M. (2023). Targeting DNA damage response pathways in cancer. *Nature Reviews Cancer*.

